# Economic Complexity Based Recommendation Enhance the Efficiency of the Belt and Road Initiative

**DOI:** 10.3390/e20090718

**Published:** 2018-09-19

**Authors:** Hao Liao, Xiao-Min Huang, Alexandre Vidmer, Yi-Cheng Zhang, Ming-Yang Zhou

**Affiliations:** 1 National Engineering Laboratory on Big data Application on Improving Government Governance Capabilities, Guangdong Province Key Laboratory of Popular High Performance Computers, College of Computer Science and Software Engineering, Shenzhen University, Shenzhen 518060, China; 2 Department of Physics, University of Fribourg, Chemin du Musée 3, 1700 Fribourg, Switzerland

**Keywords:** economic complexity metrics, the Belt and Road initiative, recommendation algorithm

## Abstract

The Belt and Road initiative (BRI) was announced in 2013 by the Chinese government. Its goal is to promote the cooperation between European and Asian countries, as well as enhancing the trust between members and unifying the market. Since its creation, more and more developing countries are joining the initiative. Based on the geographical location characteristics of the countries in this initiative, we propose an improvement of a popular recommendation algorithm that includes geographic location information. This recommendation algorithm is able to make suitable recommendations of products for countries in the BRI. Then, *Fitness and Complexity* metrics are used to evaluate the impact of the recommendation results and measure the country’s competitiveness. The aim of this work is to provide countries’ insights on the ideal development direction. By following the recommendations, the countries can quickly increase their international competitiveness.

## 1. Introduction

In September 2013, the Chinese government proposed to jointly build the *Silk Road Economic Belt* and the *21st-Century Maritime Silk Road*, which are known as the *Belt and Road initiative* (BRI). This policy was introduced during the government visits to Central Asia and Southeast Asian countries [1,2]. Now, the Belt and Road covers countries and regions in Central Asia, the North and West Asia, the Indian Ocean, and the Mediterranean area. The BRI is an open international economic cooperation network that includes more than 65 countries with 65% of the world population and 40% of the GDP in 2017. In order to expand the land and maritime transport links between China and Europe, this initiative links people all over the world and provides opportunities for global trade [3,4]. For China, the BRI will help redistribute the excess production and reduce the imbalance regarding the development of China’s regions [5]. Another important aim is to develop the economy through enhanced cooperation between countries: since the financial crisis, the global economic situation has been unfavorable. It is then imperative to build joint economic growth regions on a global scale [6]. Exports are one of the most important aspects of the BRI. Studying the characteristics of countries have great significance: both for the development of economic prosperity and to promote the regional economic cooperation. The trade potential between Asian and European countries is tremendous and the promotion of trade facilitation can further expand the trade volume.

Several studies propose modeling the characteristics of international complex networks [7,8]. In References [9,10], the authors study the trade frictions between rich and poor countries and identify the main topological features that are responsible for the difference between rich and poor countries in the international trade network. Ranking countries regarding to their economic performance is an active field [11,12]. These two studies rely solely on the topological structure of the international trade. It contrasts with usual studies, as most studies revolve around the use of various economic models to analyze the characteristics or development of international trade networks [13,14,15]. Research on the international trade network is still moving towards an objective approach, which aims to analyze the data in a data-centric way. For individual countries, one of the important pieces of information is to know where to invest and what can lead the country towards a better economic development.

In order to speed up its development, a country needs an export basket with a high diversity [16]. This work showed that countries tend to develop products that are similar to the ones they are currently exporting. This way of development is similar to the ones used in the recommender systems. Due to this particularity, the recommender system can filter large amounts of information and recommend suitable commodities for the country according to the previous export records [17,18]. Another important feature of the international trade is the geography location [19,20]. Countries of the BRI can be divided according to the region; therefore, it is very important to design an algorithm that recommend goods to the country according to their geographical location. However, up to now, there are only a few studies on recommendation algorithms with geographical locations especially in international trade networks [21].

In this work, we start by showing a way to improve the diffusion recommendation algorithm. According to the properties mentioned above, we combine geographic information to diffusion recommendation algorithms. The result shows that the improved algorithm is a better tool to build more accurate and useful recommendations. Then, we focus on the impact of the recommendations on the economic complexity of countries in the BRI. We show with an experimental evaluation that, if countries follow our recommendations, their growth and their ranking is greatly improved. This work can help countries in different regions understand the combination of export commodities that suit their region. In this way, exporting countries will clarify the direction of the country’s exports and expand the export’s basket to enhance the country’s economic strength.

## 2. Materials and Methods

### 2.1. International Trade Network

The goal of this work is to design a recommendation method apt for economic systems, especially for the countries along the Belt and Road initiative. The network of interest here is the countries–products bipartite network, in which the products correspond to exported goods. One main difference from usual social networks—for instance, the movies network—is that the countries have limited resources. They cannot produce everything and some products are too complex to be produced for a given country. The other difference is that the network is not growing, but ‘evolving’, meaning that the number of links is more or less constant, as some links are removed from the network if the country is no longer considered as a producer (see RCA (Revealed Comparative Advantage) below).

In practice, we perform the recommendation for one year in the future. The training set is the data available at year *T*, as we also use the previous years to perform the recommendation. The test set on which we evaluate the performance of our algorithm is the state of the network at year T+1. In our dataset, *T* starts at 2002 and end in 2014.

The international trade dataset we use was cleaned using *harmonization* techniques. The products are first classified into categories (6-digit Harmonized System), and the trade volume is then adjusted depending on the quantity of the reports of the individual countries. For additional details on the procedure, see Reference [22]. The original data contained 253 countries and 786 commodities from 2001 to 2015 years. We further cleaned up the data and removed the countries that had no export data. The final international trade network is composed by 192 countries and 786 commodities. For each country, the flow of export is given in USD for every product and for each year (from 2001 to 2015).

### 2.2. RCA

In the international trade network, some countries export tiny amount of goods, and so cannot be considered as an important exporter for this good. It is then necessary to further determine whether the country *i* can be considered as an exporter of commodity α. Intuitively, country *i* should contribute to a significant proportion to the export volume among all the countries which are exporting α. In order to judge if a country has enough export in a given product to be considered as a producer, we use the Revealed Comparative Advantage (RCA) [23]:

(1) $$RCA_{i\alpha }=\frac{\frac{q_{i\alpha }}{\sum_{j=1}^{N}q_{j\alpha }}}{\frac{\sum_{\beta =1}^{M}q_{i\beta }}{\sum_{\beta =1,j=1}^{\beta =M,j=N}q_{j\beta }}},$$

where $q_{i\alpha }$ is the export of product α by country *i*. The country *i* is considered as a producer of product α only if $RCA_{i\alpha }>1$.

### 2.3. Diffusion Based Recommender Algorithms

#### 2.3.1. Standard

The HybridS algorithm was inspired from the physical process of diffusion [24]. The basic idea behind the algorithm is that, if we let resources diffuse in a network, the resource will propagate to similar nodes. In other words, we use diffusion to create a similarity score.

The HybridS method is composed of two steps of diffusion. The first step is the diffusion of resources from items to users

(2) $$f_{ij}=\sum_{\alpha =1}^{M}\frac{a_{i\alpha }a_{j\alpha }}{k_{\alpha }^{\lambda }k_{j}^{1-\lambda }},$$

where λ is an adjustable parameter, $a_{i\alpha }$ is 1 if country *i* produces item α, 0 if not. $k_{i}$ and $k_{\alpha }$ are the degree of item *i* and itemize α, respectively, *M* is the number of items. $f_{ij}$ is used in the second step of diffusion, which diffuses resources back to the items:

(3) $$f_{i\beta }=\sum_{j=1}^{N}\frac{a_{j\beta }f_{ij}}{k_{j}^{\lambda }k_{\beta }^{1-\lambda }},$$

where *N* is the number of countries and $f_{i\beta }$ is the recommendation score of item β for user *i*. These scores are then sorted from the biggest to lowest value and become the recommendation list for user *i*, in which we remove already collected items. When λ=1, the diffusion process is equivalent to a Probabilistic spreading, hence the name ProbS, and, when λ=0, it is equivalent to the Heat diffusion process, hence the name HeatS.

The time dependent term includes the dynamical features of the network growth as follows [25]:

(4) $$\Delta k{}_{\alpha }^{\prime }\left(t,\tau \right)=\Delta k_{\alpha }\left(t,\tau \right)+\varepsilon k_{\alpha }\left(t\right),$$

where $\Delta k_{\alpha }\left(t,\tau \right)=k_{\alpha }\left(t\right)-k_{\alpha }\left(t-\tau \right)$ is the increased degree of item within the time window length τ. The parameter ϵ is present to ensure that $\Delta k{}_{\alpha }^{\prime }\left(t,\tau \right)$ is non zero, but should be small enough to not change significantly the result. In this work, we fixed ϵ = 1 × 10${}^{-5}$. The TProbS is combined with the ProbS method with the Degree Increase method. On top of the diffusion process, a time bias is added in the TProbS algorithm in order to enhance the performance of the recommendation [26]. Similarly, the THybridS combines the Hybrid method with a Degree Increase method.

#### 2.3.2. Preferential Diffusion

As shown in [27], we can enhance the recommendation by exponentiating the scores after the second step of diffusion so the final step of diffusion becomes

(5) $$f_{i\beta }=\sum_{j=1}^{N}\frac{a_{j\beta }f_{ij}^{\theta }}{k_{j}^{\lambda }k_{\beta }^{1-\lambda }}.$$

When the Preferential diffusion scheme is used, we add a *PD* prefix to the name of the diffusion method (e.g., THybridS become PD-THybridS).

#### 2.3.3. Geography

Countries that are closer to each other tend to compete on the same type of goods [28]. Using this information, we use the distance from one country to another in the second diffusion step:

(6) $$f_{ij}=\sum_{\alpha =1}^{M}\frac{a_{i\alpha }a_{j\alpha }e^{D(i,j)\theta }}{k_{\alpha }^{\lambda }k_{j}^{1-\lambda }}.$$

Compared to the standard diffusion algorithm, we replace $f_{i\beta }$ by $e^{D(i,j)\theta }$ in the second diffusion step, where D(i,j) is the distance of country *i* and *j* normalized with the distance to the closest country of *i* (expect itself). When the Geography diffusion scheme is used, we add a *Geo* prefix to the diffusion method (e.g., THybridS become Geo-THybridS). All four of these versions of diffusion were improved by the use of geography information.

### 2.4. Recall

By the use of the recommendation method, we predict the future production for each country. In order to evaluate the efficiency of the algorithm, we use the precision *P* as a measure, which is the ratio of good guess in the top *L* recommendation list and the length of the list itself (*L*). In the same way, the recall R(L) is the ratio of the good guess in the top-*L* recommendation list and the number of new items in the export list of each country.

### 2.5. Fitness and Complexity Metrics

The Fitness and Complexity metrics were proposed to measure the competitiveness of countries and exported production [29]. The algorithm points out that the complexity of goods should be affected by countries with few exports because powerful countries export almost all goods, and the complexity of goods cannot be obtained from their export behavior [30,31]. In this way, the Fitness and Complexity metrics are implemented in a nonlinear iterative and the convergence of the algorithm is studied in Reference [32]. Fitness and Complexity metrics show the superiority of other economic indicators in [33]. We define the countries fitness $F_{i}$ and item complexity $Q_{\alpha }$ by an iterative method:

(7) $$\left\{\begin{array}{c} {\tilde{F}}_{i}^{\left(N\right)}=\sum_{\alpha =1}^{M}a_{i\alpha }Q_{\alpha }^{\left(N-1\right)},\\ {\tilde{Q}}_{\alpha }^{\left(N\right)}=\frac{1}{\sum_{i=1}^{N}a_{i\alpha }\frac{1}{F_{i}^{\left(N-1\right)}}}, \end{array}\right.$$

where the subscript *N* denotes the iteration. All values for fitness and complexity are initialized at 1 for iteration 0. At each iteration, we also normalize fitness and complexity:

(8) $$\left\{\begin{array}{c} F_{i}^{\left(N\right)}=\frac{{\tilde{F}}_{i}}{{\langle {\tilde{F}}_{i}\rangle }_{i}},\\ Q_{\alpha }^{\left(N\right)}=\frac{{\tilde{Q}}_{\alpha }}{{\langle {\tilde{Q}}_{\alpha }\rangle }_{\alpha }}. \end{array}\right.$$

For the algorithm, we use the stopping conditions that were studied and described in Reference [32].

## 3. Results

### 3.1. Comparison of Recommendation Algorithms

Recommendation algorithms are designed to recommend items to users. In addition, they can also be used to predict what the users will do in the future. In our case, we use the recommendation systems as a predictive tools for countries and their production. Based on the production of a country at year *T*, the goal is to predict its production at year T+1. Then, the recommendation results are evaluated with Precision and Recall, two metrics to assess the accuracy of the prediction. In this work, we use a recommendation algorithm inspired from the physical process of diffusion. This algorithm is named the hybrid diffusion method, as it can be hybridized thanks to a free parameter λ, which requires being adjusted depending on the dataset. Note that, in some cases, optimizing the additional parameter and λ could improve further the results, at the expense of additional computation.

Additionally, we add two modifications on the hybrid diffusion method: Preferential diffusion and Geography diffusion. The aim of Preferential diffusion is to avoid recommending the same objects to all the users. This algorithm is mitigated by a parameter θ, θ=0 means no preferential diffusion, and the higher the parameter is, the more we force the algorithm to recommend objects that have very few connections. The Geography method uses geographical positions of countries to gives more weight to countries that are closer. The strength of this weight can also be tuned with a parameter θ. If θ>0, the algorithm gives more weight to distant countries, while θ<0 gives more weight to closely located countries. See Section 2 for additional details on algorithms and metrics.

The results for Precision and Recall are shown in Table 1 on page 5. It is clear that the Preferential diffusion and Geography modification improve the accuracy. The best overall result is obtained coupled with PD-ProbS. The value of the θ parameter for Preferential diffusion is interesting. In social networks such as Netflix or Movielens, the optimal value of the parameter is 1.9 and 2.6, respectively [27]. However, in the international trade, this value is only 2.20 in PD-ProbS. Our interpretation of this is that, while countries tends to replicate the development path of other countries, the effect is less pronounced than in social networks: if every country is producing the same goods, the international trade will become quickly saturated with this product. If the market is saturated with a product, it becomes harder and harder to make profit on it.

The Geography based algorithm improves the Recall even further. The highest accuracy is achieved with θ=−1.80. The distance are normalized with respect to the closest country. This means that countries tend to export goods similar to neighboring countries, and not just the closest country. This obviously make sense as the available resources and available knowledge of neighbors countries can be very similar.

In Reference [26], the recall was around 14%. The main difference is that performing the prediction for the following year, while, in the other study, the prediction was performed for a period of five years. We note that the Geography and Preferential modifications are really able to grasp information on the short time evolution of countries.

**Figure 1:**
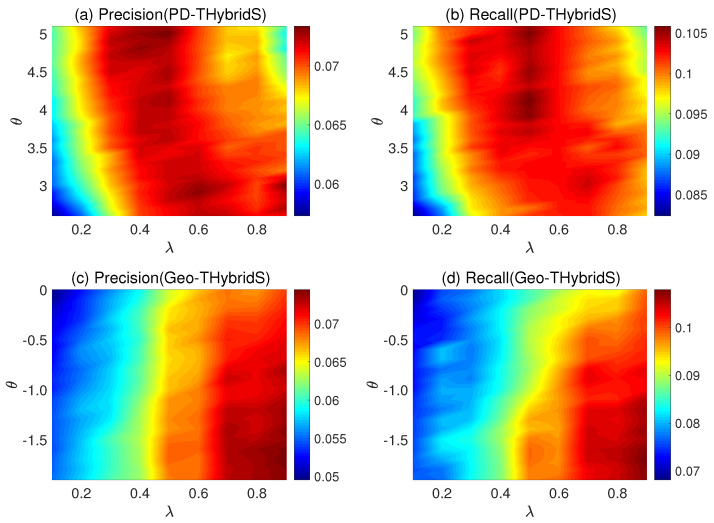
(**a**,**b**) show the precision and recall with varying parameter λ and θ of PD-THybridS. Similarly, the precision and recall of Geo-THybridS are show in the panel (**c**) and panel (**d**).

For PD-THybridS algorithm and Geo-THybridS, we compare the performance of the algorithm as a function of the parameters λ and θ. The results are shown in Figure 1 on page 6. For the countries and products data, we found λ=0.50 and λ=0.90 to be the values optimizing the recall for PD-THybridS and Geo-THybridS, respectively. These values are used for the rest of the work.

### 3.2. The Relationship between Fitness and Links

**Figure 2:**
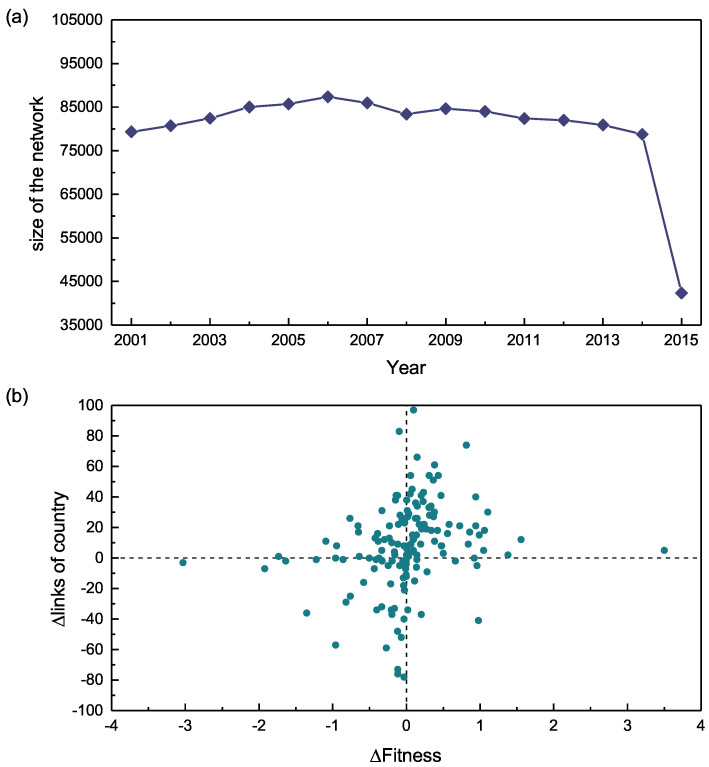
(**a**) shows the evolution of the network size from 2001 to 2015; (**b**) is the relationship between Δlinks of country and the Δfitness value of country. Each dot represents a specific country.

The countries’ fitness value is determined by quantity and complexity of exported commodities. Obviously, the countries with high fitness tend to enhance their exports complexity or expand the export’s basket. The size of the network is nearly constant over years for the whole international trade network, as we can find in Figure 2 on page 7 (panel (a)). Because of the data incompleteness in 2015, we choose sample trading data in 2001 and 2014, respectively, in Figure 2 on page 7 (panel (b)) and we illustrate that the evolving quantity change of export commodities has a positive correlation with the change of the specific fitness value of the country. The Spearman correlation coefficient is r=0.39.

The fitness value of the country will improve with the increase of export commodities. For some competitive countries, they can rapidly upgrade their fitness by exporting a small amount of high complexity commodities such as Switzerland exports of new medicine products. On the contrary, countries with poor productive capacity export large quantities of products that are easy to produce, and the value of fitness is only slightly changed, such as Dominica export Cotton fabrics. This figure clearly shows that the fitness value is not only related to the number of exports but also to the complexity of the exported commodities.

### 3.3. Complexity Metric

Recommending complex items can be a sign of a good algorithm; however, it must fulfill some conditions. The complexity of recommended items should depend on the fitness of the countries. We define the average rank complexity of the *L*, first items in the recommendation list C(L). This factor is normalized by the number of items and the length of the recommendation list, a low score indicates that we recommend complex items in average, and a high one indicates that we recommend less complex items. We defined this metric in order to measure the quality of our recommendation.

**Figure 3:**
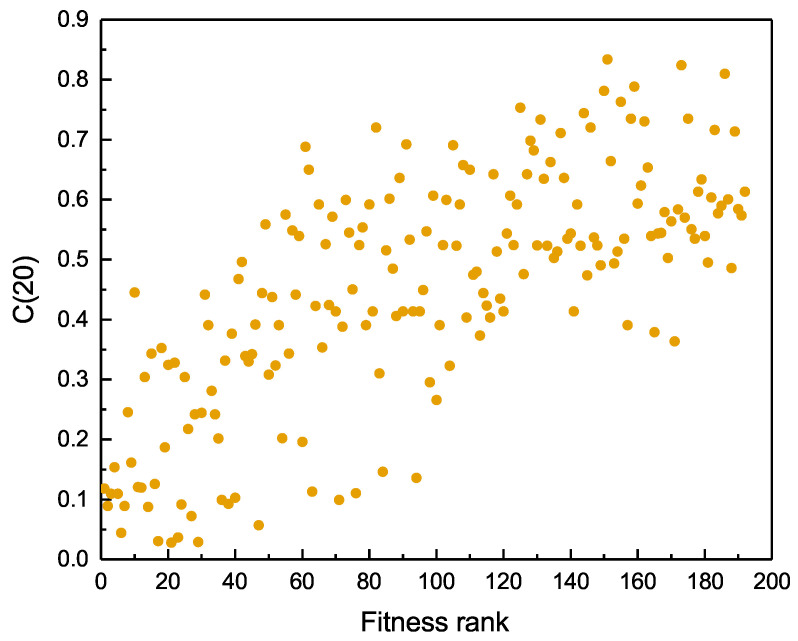
The relationship between the countries’ fitness rank and its average rank complexity of the top 20 recommended commodities. C(20) means that the length of the recommendation list is set to 20. The complexity is the average of items’ complexity in the Geo-THybridS recommendation list. Each dot represents a specific country.

As shown in Figure 3 on page 8, the complexity of the recommended products is positively related to the fitness ranking of the country. The result is very clear—that the recommendation makes a reasonable prediction, the higher ranked countries have high productivity, which is capable of producing more complex goods to improve the country’s comprehensive strength. However, countries with a low fitness rank are limited in terms of production capacity and export strength. They need to gradually improve their fitness value through exporting easily produced commodities. However, even for the countries with the lowest ranking, the recommendation algorithm does not recommend the least complex items. The result demonstrates that the recommendation algorithm can help high fitness countries. More importantly, the low fitness countries can design a better production policy to enhance their countries fitness. Just as the saying goes, “Rome was not built in a day”—any great achievement isn’t done within days or months.

### 3.4. The Belt and Road Initiative

The countries along the Belt and Road initiative are classified according to the region. The regions are specifically divided into: one country of East Asia, 10 countries of Association of Southeast Asian Nations (ASEAN), 18 countries of West Asia, eight countries of South Asia, five countries of Central Asia, seven countries of Commonwealth of Independent States (CIS) and 16 countries of Central and Eastern Europe. According to the characteristics of this initiative and the result shown in Figure 3 on page 8, we see that the Geo-THybridS algorithm is indeed recommending lower complexity products to lower fitness countries, and vice versa. We treat every country in a region as a category and count the number of the top 5 recommended items in each region. The most recommended commodities in each region are shown in Table 2 on page 9.

#### Fitness Evolution

**Figure 4:**
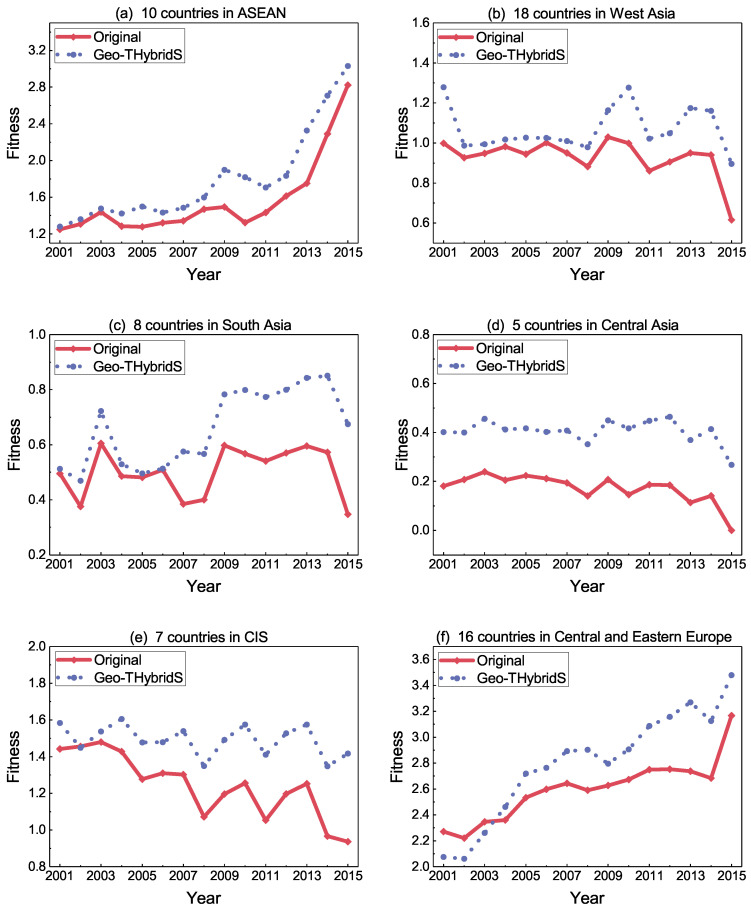
(**a**–**f**) shows the evolution of fitness values in various regions from 2001 to 2015, respectively. Among them, the fitness value is the average fitness of all countries in the specific region.

Since the countries along the Belt and Road initiative are divided according to the region, we use the evolution of the national fitness to reveal the results of the Geo-THybridS algorithm. We want to know, if every country in the same region produces the recommended products, how the fitness will evolve. We add the new links of country-product pairs to the international trade network and keep other conditions unchanged. The fitness values are then recomputed with the modified network. The comparison of simulated fitness values (Geo-THybrid) with the original fitness is shown in Figure 4 on page 10.

The interesting phenomenon is that the trend of the original fitness curve and the Geo-THybrid fitness curve is quite similar in each region within 2001 to 2015. In other words, in most cases, the newly added products only increase the value of fitness and there is no obvious effect on the trend of the original fitness curve. We also find that the original fitness decline in the whole region is mainly due to the obvious decrease in some countries’ fitness. Thus, we decided to add 20 goods in the network, with a lower value, the effect on the network is not noticeable, and a higher value would disturb the network too much. The exact number is not crucial as it does not change the tendency of results. Similarly, when the original fitness rise, the added goods could further increase the value of fitness. However, the exports from various countries are changed every year, resulting in the complexity of the products not being static, which explains why the evolution of the two curves is not parallel. The effect of the recommendation algorithm on countries in different regions is diverse—for example, the region of the 16 countries in Central and Eastern Europe (panel f) whose fitness value obviously improves when they adapt the recommendation products list in 2009–2015. The main reason is that, for the higher original fitness countries, the recommended products are more complex, which can significantly improve the fitness value of their countries. On the contrary, the fitness of the five countries in Central Asia with the additional 20 recommended products has about 0.2 improvement of their fitness. We can identify the fact that middle-tier fitness countries can gain more fitness value through the recommendation method, and the low-tier fitness countries can enhance their capacity of production and fitness among the similar ranked countries. Although there is not much consistency among commodities in each region Table 2 on page 9, the complexity of products can be seen in different regions. In general, the additional 20 recommended products does not change the fluctuation of the region fitness and it only can improve the value of fitness. It shows that the recommended results have diverse effects on different economic performance regions.

**Figure 5:**
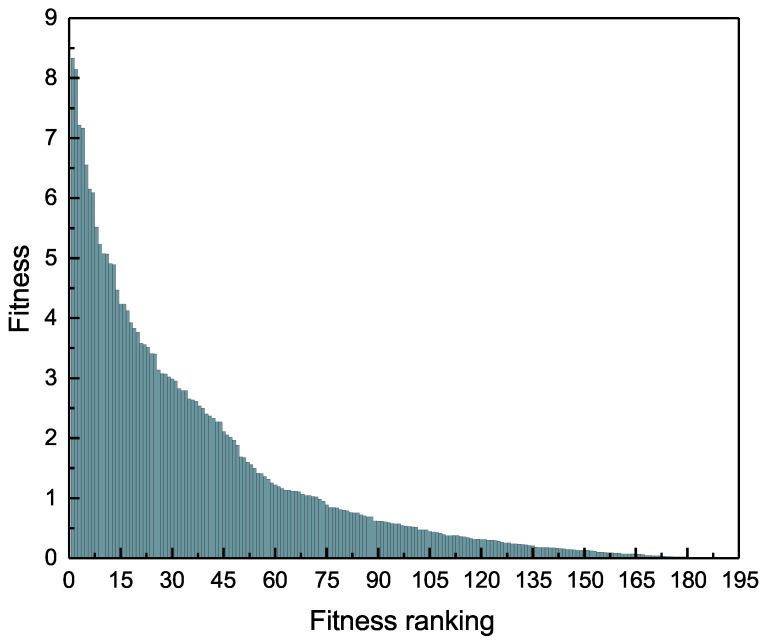
The distribution of the original fitness value of the country.

Figure 5 on page 11 shows the distribution of the original fitness value. We find that the gap between the top ranked countries fitness is very obvious, but those countries ranked lower, and the disparity in fitness value is smaller. For instance, the fitness values of the top 15 countries differed by approximately 4; however, the percentage of countries with a fitness value less than 1 is more than 50%. This distribution can be analyzed together with Figure 4 on page 10. It shows that, for countries with lower fitness ranking, perhaps the increase of fitness value is not very obvious, but it has a great effect on the ranking of the countries fitness. These sort of countries can try to export low-complexity commodities to improve fitness rank. On the contrary, the top ranked countries are fiercely competitive and they must produce more complex goods to improve the country’s ranking. Indeed, it was shown in various model studies that the top ranking countries are the ones focusing on quality/complex goods [34,35,36].

## 4. Discussion

The work can be divided in two main parts: the prediction of countries’ future exports and the fitness evolution of countries separated in different regions. We showed that, by taking into account the geographical information, we were able to greatly improve the accuracy of the prediction. This leads to two important points. First, our algorithm is able to predict the evolution of countries with relatively high accuracy. Secondly, the evolution of countries is strongly influenced by their region.

The Belt and Road initiative promotes the economic and trade between the Member States. Therefore, we chose this to analyze in a quantitative way the economic benefit of the countries’ part of this initiative. Knowing that the region plays a major role in the BRI, we divided the dataset in seven different regions in order to analyze the evolution of their fitness. We gave recommendations for each country, and we simulated the evolution of fitness in a hypothetical case where the countries would have followed our recommendations. We saw that some regions were more prone to a stronger fitness evolution, while some of our recommendations had less impact. However, it is evident that our proposed method can only be considered as supportive information but not decisive information.

To conclude, we proposed a way to help policy makers decide which path to go on regarding the evolution of their countries. Our algorithm has very few parameters and is thus meant as a side tool to suggest evolution paths. To go further, it would be interesting to see what could be the impact of our recommendation algorithm in a more traditional economics forecast.

## Figures and Tables

**Table 1:** A comparison of the precision and recall of the three algorithms. The θ parameters for Preferential diffusion and Geography algorithms are the one maximizing Precision and Recall. The bold data means the best performance in these set of methods.

Algorithm	θ	P(20)	R(20)
ProbS	-	**0.067**	0.094
HeatS	-	0.063	0.095
TprobS	-	0.067	**0.096**
THybridS	-	0.066	0.095
PD-ProbS	2.20	**0.076**	**0.108**
PD-HeatS	2.10	0.065	0.099
PD-TprobS	1.80	0.070	0.098
PD-THybridS	3.90	0.072	0.104
Geo-ProbS	−1.90	**0.075**	0.106
Geo-HeatS	−0.50	0.067	0.100
Geo-TprobS	−2.00	0.074	0.104
Geo-THybridS	−1.80	**0.075**	**0.108**

**Table 2:** A presentation about the most recommended products in different regions.

Country	Recommended Products
1 country of East Asia	Tugs, special purpose vessels and floating structures
	Oil seeds and oleaginous fruits
	Sheep and lamb skin without the wool, raw
	Yarn of regenerated fibres, put up for retail sale
	Imitation jewellery
10 countries of ASEAN	Crystals, and parts of electronic components
	Base metals and cermets, unwrought
	Sheep and lamb skin with the wool on
	Ores and concentrates of other non-ferrous base metals
	Briquettes, ovoids, from coal, lignite or peat
18 countries of West Asia	Chemical elements
	Poultry, live
	Groundnut (peanut) oil
	Discontinuous synthetic fibres
	Refined sugar etc
8 countries of South Asia	Natural honey
	Sawlogs and veneer logs, of non-coniferous species
	Cotton linters
	Distilled alcoholic beverages
	cellulosic pulps
5 countries of Central Asia	Wood packing cases, boxes, cases, crates, etc., complete
	Builders’ carpentry and joinery (including prefabricated)
	Fabrics woven of sheep’s or lambs’ wool or of fine hair
	Glass, etc, surface-ground, but no further worked
	Railway or tramway sleepers (ties) of wood
7 countries of CIS	Copper ore and concentrates; copper matte; cement copper
	Other natural abrasives
	Cigarettes
	Animals oils, fats and greases
	Vegetable textile fibres
16 countries of Central and Eastern Europe	Tugs, special purpose vessels and floating structures
	Parts of and accessories for musical instruments; metronomes
	Anti-knock preparation, anti-corrosive; viscosity improvers
	Batteries and electric accumulators, and parts thereof
	Precious and semi-precious stones, not mounted, set or strung
